# Supplementary material for: Differentiation signals induce APOBEC3A expression via GRHL3 in squamous epithelia and squamous cell carcinoma
Source: EMBO J. 2024 Nov 15;44(1):1–29. doi: 10.1038/s44318-024-00298-9 (PMC11696371; doi:10.1038/s44318-024-00298-9)
Supplement: Supplementary file 7 — Appendix [file 44318_2024_298_MOESM7_ESM.pdf]

Appendix to ‘Differentiation signals induce *APOBEC3A* expression via GRHL3 in squamous epithelia and squamous cell carcinoma’

Page	Appendix Figure
2	S1: *APOBEC3A* is predominantly expressed in differentiating (‘epi-stratified’) oesophageal epithelial cells.
3	S2: Effect of intracellular signalling pathway inhibition on gene expression and cell cycle profile in NIKS.
4	S3: Binary scoring of GRHL3 activity in proliferating, differentiating and terminally differentiated clusters in healthy tonsil epithelium.
5	S4: *ELF3* is induced under the same conditions as *APOBEC3A* in cultured keratinocytes.
6	S5: *GHRL3* knockdown does not affect levels of the proliferation markers, *MKI67* and *MCM7* in afatinib-treated NIKS.
7	S6: *GRHL3* knockdown reduces *APOBEC3A* mRNA levels in PMA-treated NIKS and in response to growth factor withdrawal.
8	S7: GRHL3 and WDR5 bind an *APOBEC3A* enhancer and the *ELF3* promoter in differentiating NHEKs.
9	S8: Identification of malignant and non-malignant cells in head and neck tumour single cell data.
10	S9: HNSCC and ESCC display similar expression patterns of *APOBEC3A* and *APOBEC3B* to those observed in the healthy tonsil epithelium.
11	S10: Co-expression of *APOBEC3A* with differentiation and RIPK4 pathway genes and *APOBEC3B* with proliferation-related genes in the Southampton HNSCC scRNA-seq dataset.
12	S11: *APOBEC3A* and GRHL3 expression in sub-confluent, asynchronous NIKS, BICR6 and BICR22 cell lines.
13	S12: Pathway analysis of the top 100 genes co-expressed with *APOBEC3A* and *APOBEC3B* in spatial transcriptomic data.
14	S13: Deconvolution of spatial transcriptomic data showing the cell types in which *APOBEC3A* and *APOBEC3B* are expressed within each spot.
15	S14: Generation of *APOBEC3A* knockout (KO) NIKS and specific detection of APOBEC3A by UMN-13 immunohistochemistry.
16	S15: Analysis of *APOBEC3A* and *APOBEC3B* expression in HNSCC by spatial transcriptomics and immunohistochemistry.

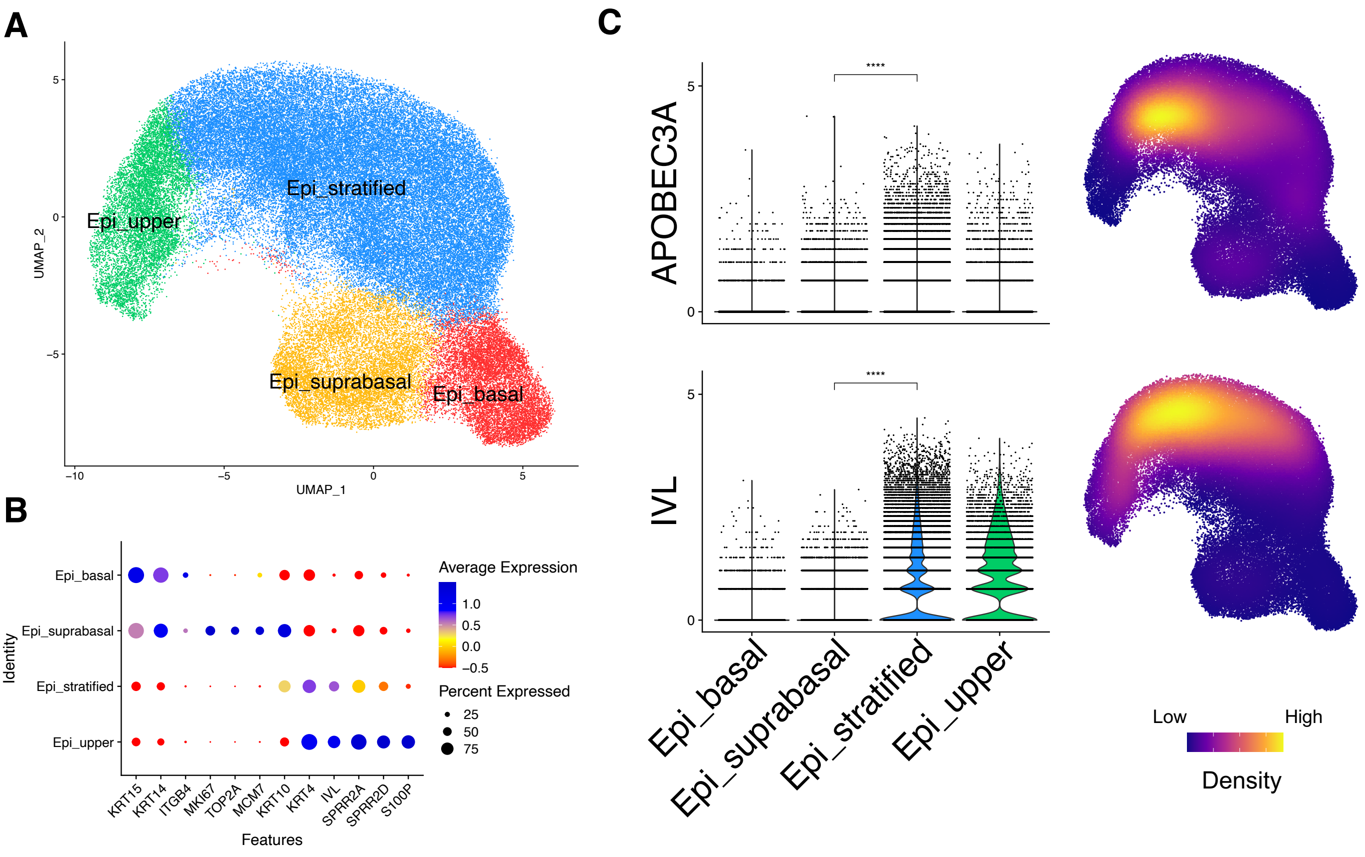

Appendix Figure S1. *APOBEC3A* is predominantly expressed in differentiating ('epi-stratified') oesophageal epithelial cells. A) UMAP projection of 80,489 oesophageal epithelial cells from healthy tissue. **B)** Dotplot of marker genes used to identify epithelial phenotypes. **C)** Violin plots of gene expression ii the oesophageal epithelial cell states (left, **** = p-value < 0.0001, Wilcoxon's Rank Sum Test) and UMAP projections of the density of gene expression in individual oesophageal epithelial cells (right).

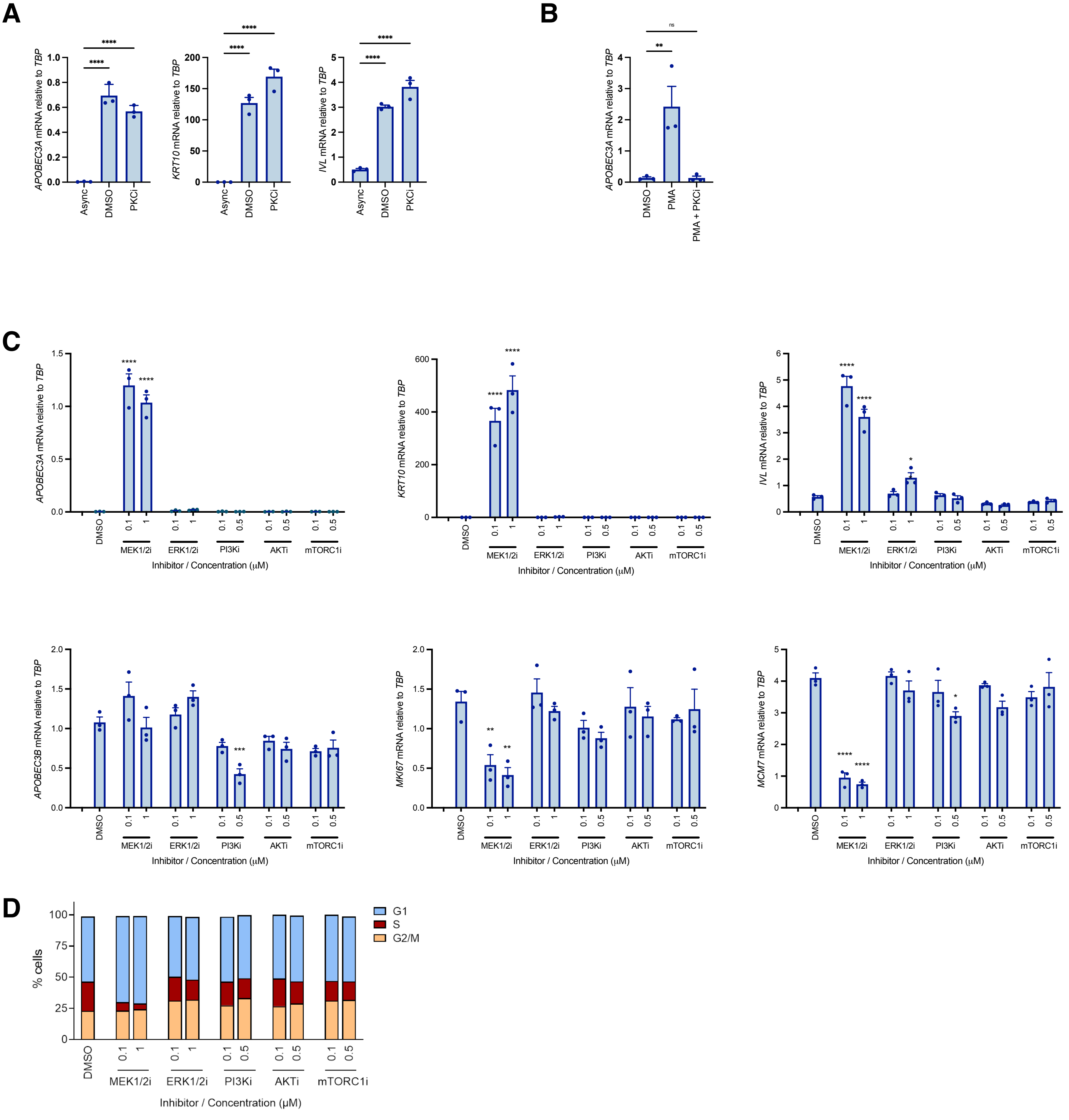

Appendix Figure S2. Effect of intracellular signalling pathway inhibition on gene expression and cell cycle profile in NIKS. A) qRT-PCR measurements of *APOBEC3A***,** *KRT10*, and *IVL* mRNA levels in asynchronous growing NIKS (‘Async’) and following 48 hours of growth factor withdrawal in the presence of vehicle control (DMSO) or PKC inhibitor (100 nM GÖ6983). **B)** qRT-PCR measurements of *APOBEC3A* mRNA levels in NIKS treated with 100 ng/ml PMA -/+ PKC inhibitor (100 nM GÖ6983) for 6 hours. **C)** qRT-PCR measurements of *APOBEC3A*, *KRT10*, *IVL* (top row), *APOBEC3B*, *MKI67* and *MCM7* (bottom row) mRNA levels in NIKS following 24 hours of treatment with vehicle control (DMSO) or inhibitors of MEK1/2 (trametinib), ERK1/2 (ravoxertinib), PI3K (pictilosib), AKT (MK2206) or mTORC1 (everolimus). N = 3, error bars represent SEM. * = p-value < 0.05; ** = p-value < 0.01; *** = p-value < 0.001; **** = p-value < 0.0001. Comparisons to proliferating (‘async’) cells in **(A)** and to DMSO-treated cells in **(B)** were performed using one-way ANOVA with Dunnett’s multiple comparisons test. **D)** cell cycle profiles of NIKS treated with inhibitors as in panel **C**.

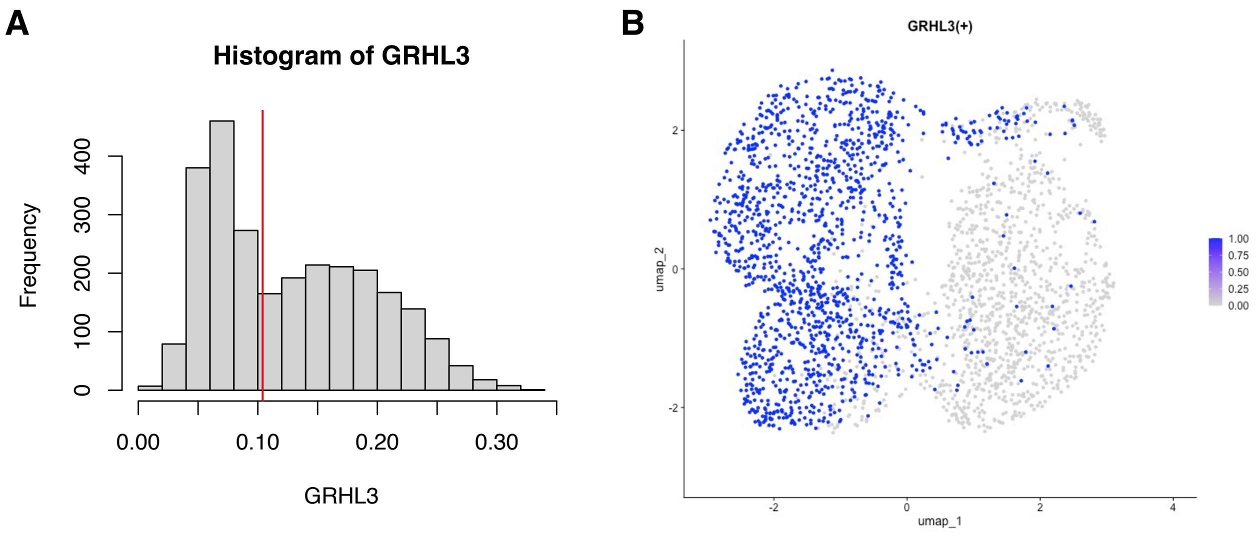

Appendix Figure S3. Binary scoring of GRHL3 activity in proliferating, differentiating and terminally differentiated clusters in healthy tonsil epithelium.

A) distribution of GRHL3 activity scores from SCENIC across the dataset. Vertical red line represents the on/off threshold.

B) UMAP showing binary activity calls for GRHL3 (blue = 'on', grey = 'off'). Accompanies Figure 4.

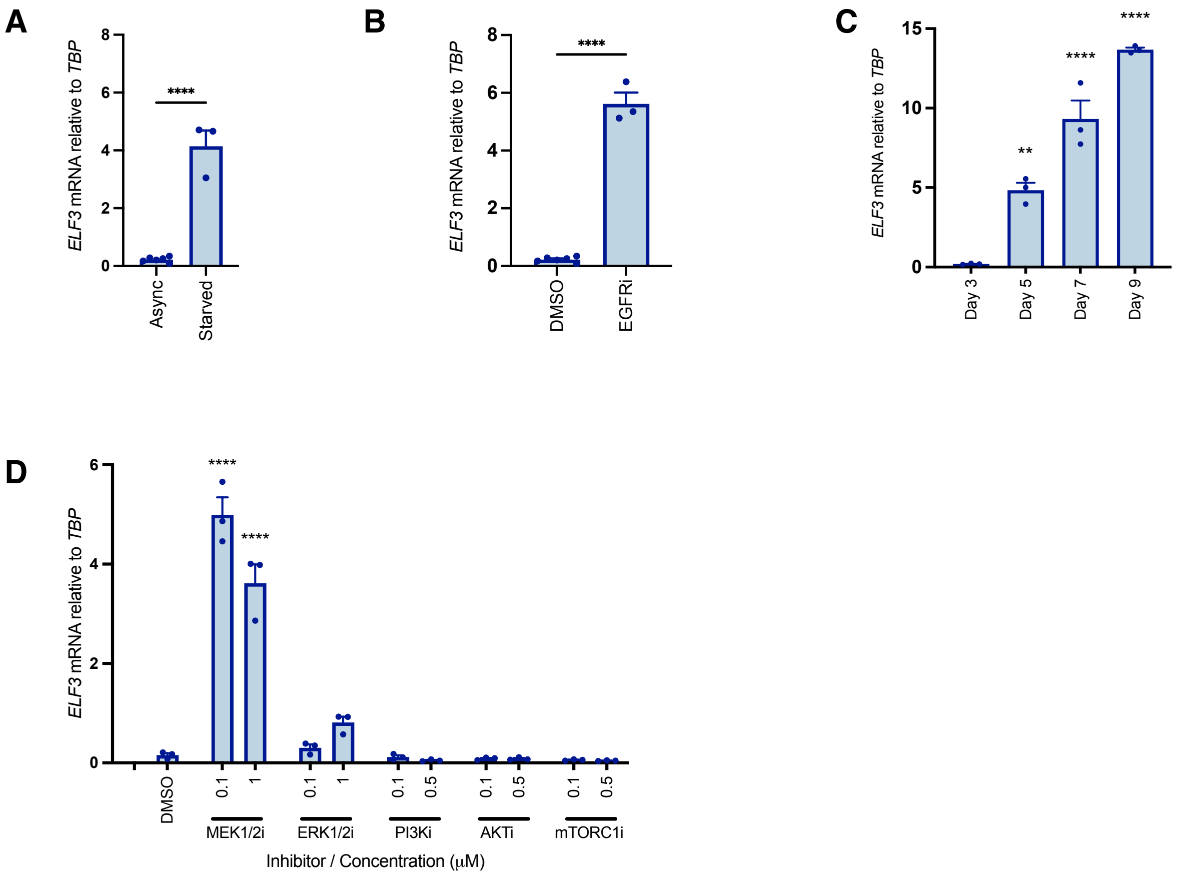

Appendix Figure S4. *ELF3* is induced under the same conditions as *APOBEC3A* in cultured keratinocytes. qRT-PCR for ELF3 in proliferating NIKS (async) or following 48 hours of growth factor and serum starvation **(A)**, 24 hours of 100 nM afatinib treatment **(B)**, growth to high cell density **(C)** or following 24 hours of treatment with inhibitors of MEK1/2 (trametinib), ERK1/2 (ravoxertinib), PI3K (pictilosib), AKT (MK2206) or mTORC1 (everolimus) **(D)**. Async cells in panels b and d were treated with vehicle control (DMSO) for 24 hours. N = 3, error bars represent SEM. ** = p-value < 0.01; **** = p-value < 0.0001. Accompanies Fig 3 A - C and F. Pairwise comparisons in **(A)** and **(B)**: two-tailed, unpaired t-tests; pairwise comparisons to Day 3 value in **(C)** and DMSO control treatment in **(D)**: one-way ANOVA with Dunnett's multiple comparisons test.

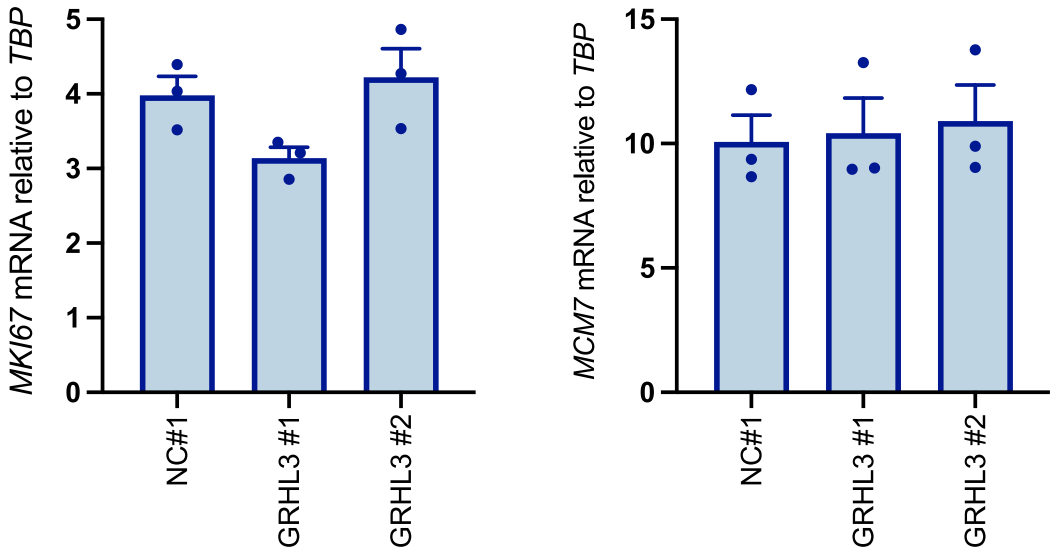

Appendix Figure S5. *GHRL3* knockdown does not affect levels of the proliferation markers, *MKI67* and *MCM7* in afatinib-treated NIKS. Histograms showing expression of *MKI67* and *MCM7,* in NIKS transfected with control siRNA (NC#1), or two different siRNAs targeting *GRHL3* as indicated. Cells were treated with 100 nM afatinib for 24 hours prior to harvesting to induce differentiation. N = 3, error bars represent SEM. Gene expression in NIKS transfected with siRNAs targeting GRHL3 was compared with control siRNA-transfected NIKS by one-way ANOVA with Dunnett's multiple comparisons test. Accompanies Fig 4E.

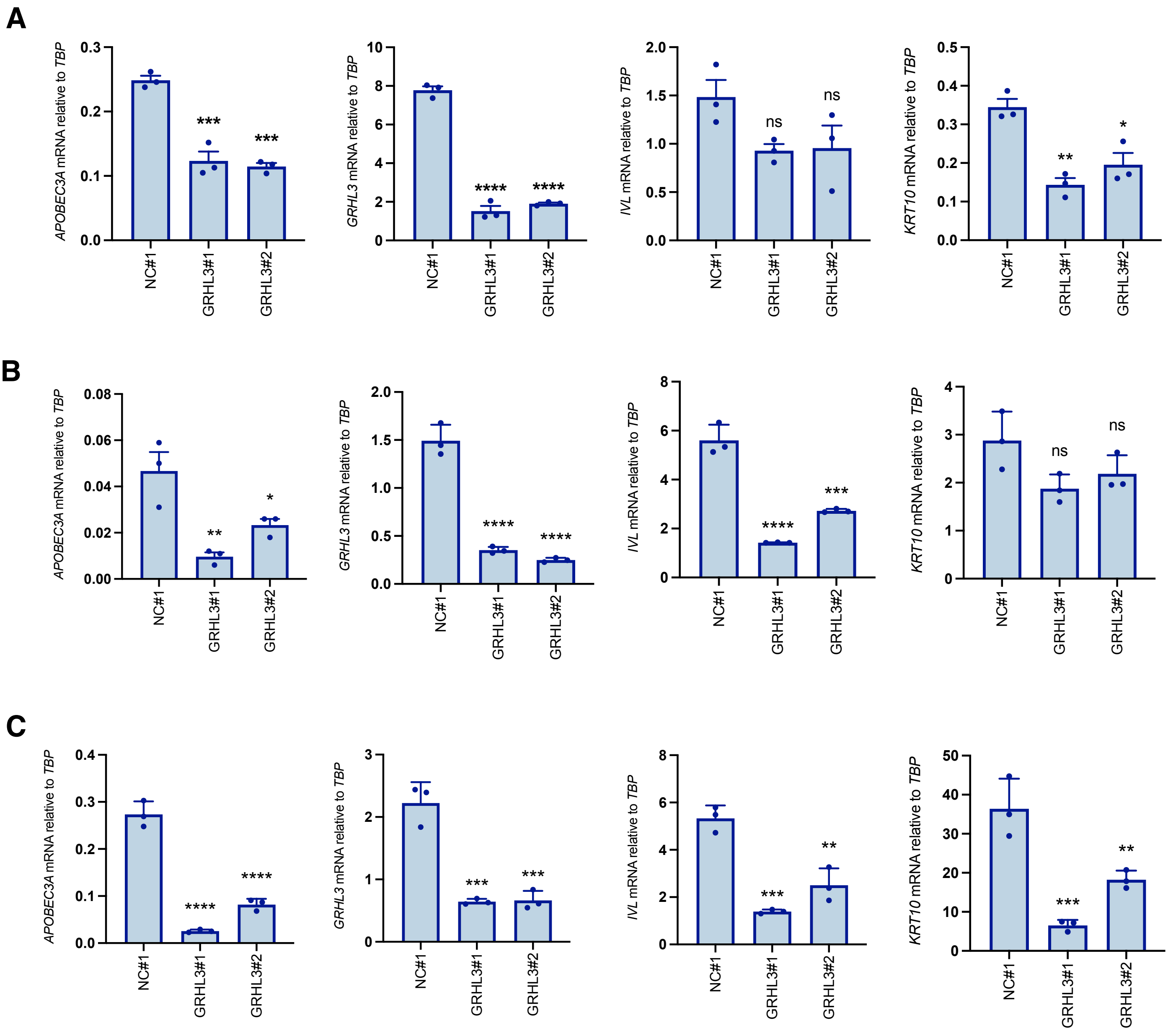

Appendix Figure S6. *GRHL3* knockdown reduces *APOBEC3A* mRNA levels in PMA-treated NIKS and in response to growth factor withdrawal. qRT-PCR measurements of *APOBEC3A*, *GRHL3*, *IVL* and *KRT10* mRNA levels in NIKS following treatment with 100ng/ml PMA for 3 hours **(A)**, withdrawal of serum and growth factors for 40h (**B**) and removal of only EGF for 40h (**C**). N = 3, error bars represent SEM. * = p-value < 0.05; ** = p-value < 0.01; *** = p-value < 0.001; **** = p-value < 0.0001. Comparisons between cells transfected with a negative control siRNA (NC#1) and with GRHL3 siRNAs were performed using one-way ANOVA with Dunnett's multiple comparisons test.

A

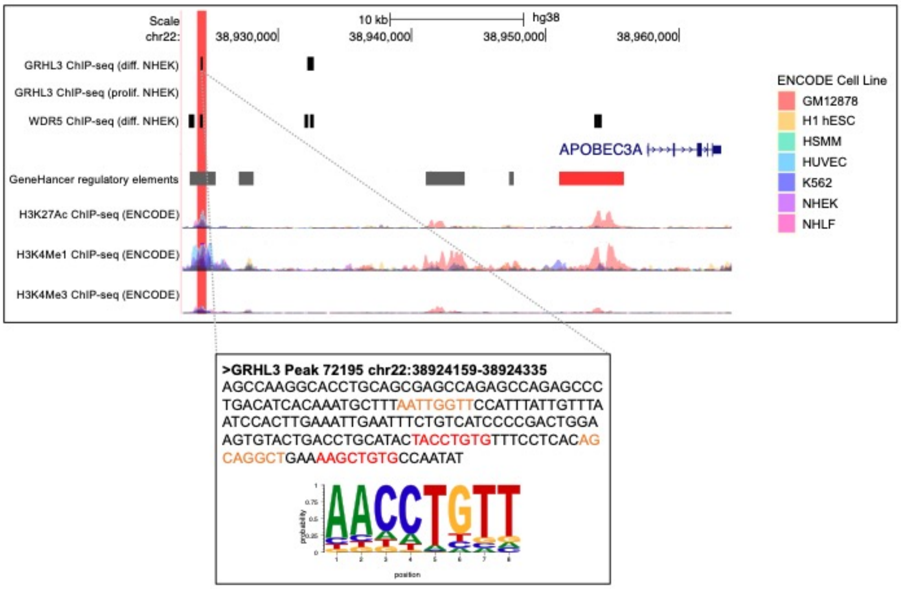

B

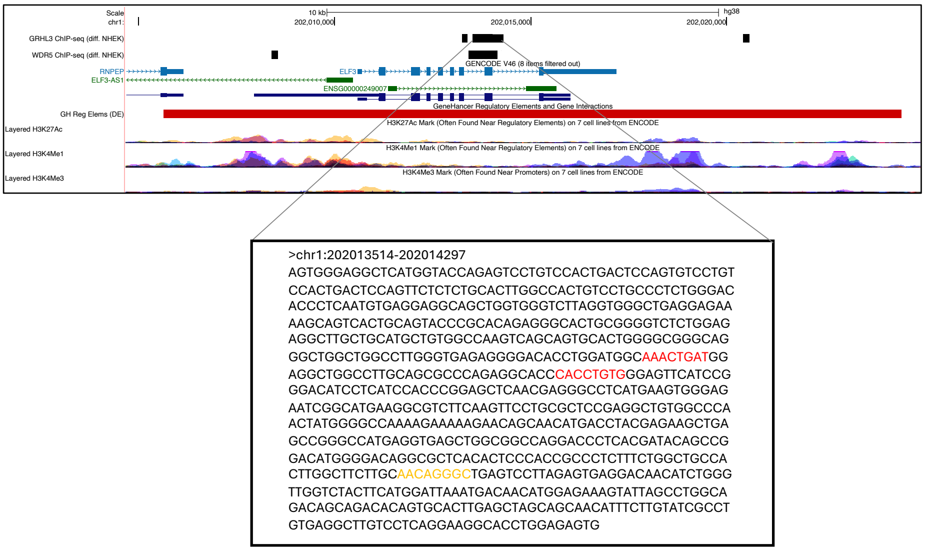

Appendix Figure S7. GRHL3 and WDR5 bind an *APOBEC3A* enhancer and the *ELF3* promoter in differentiating NHEKs. UCSC Genome browser track showing **A)** APOBEC3A gene and location of GRHL3 and WDR5 ChIP-seq peaks, and **B)** ELF3 gene and location of GRHL3 and WDR5 ChIP-seq peaks. Boxes below UCSC tracks show sequence of GRHL3 peaks and locations of the GRHL3 binding motif (red = + strand, orange = - strand). Accompanies Fig 4F.

A

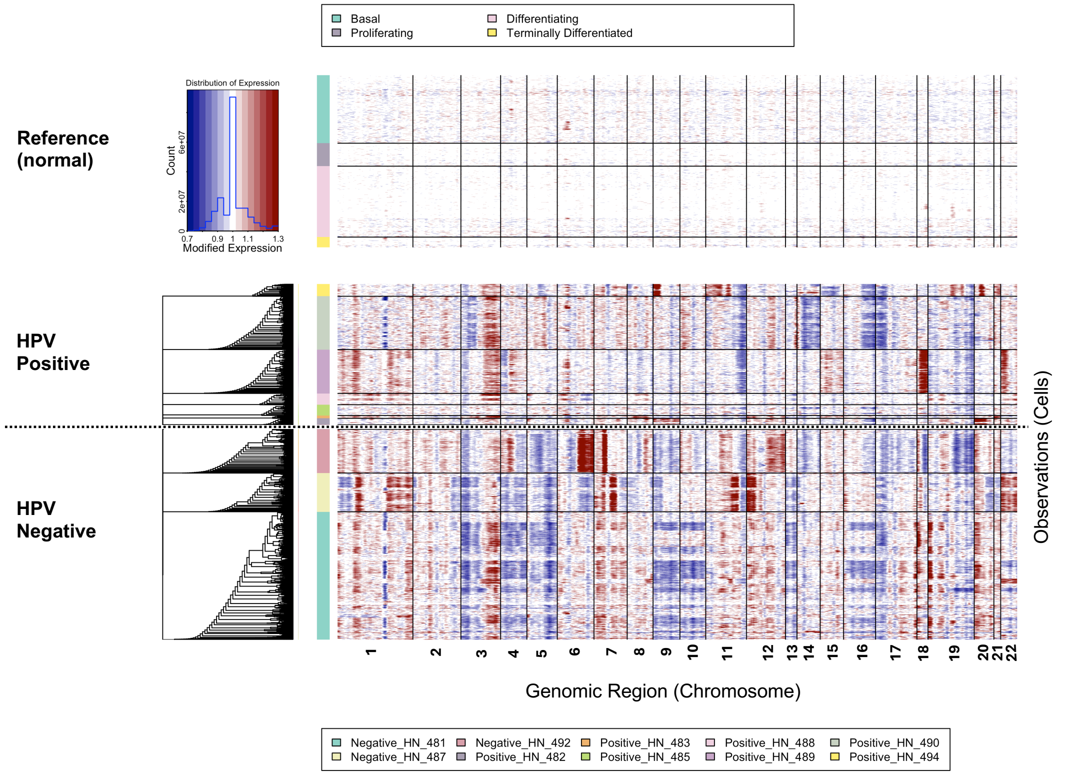

B

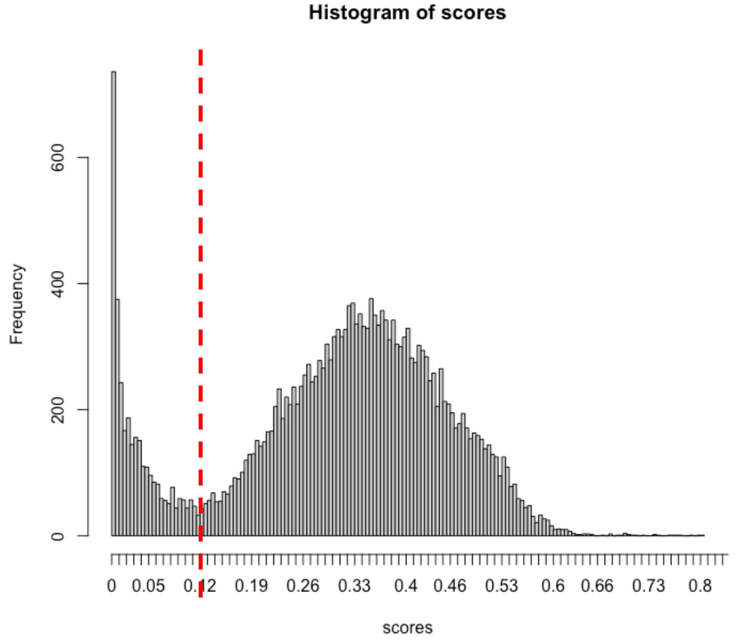

Appendix Figure S8. Identification of malignant and non-malignant cells in head and neck tumour single cell data. A) Heatmap showing inferred CNV profile for reference cells (matched normal cells; top heatmap) grouped by cell type and for tumour cells (bottom heatmap) grouped by patient. Red represents inferred copy gains, and blue copy deletions. **B)** Histogram of CNV scores calculated based on the number of genes with copy number alterations. The red dashed line illustrates the threshold for “malignant” (to the right of the line) and “non-malignant” (to the left).

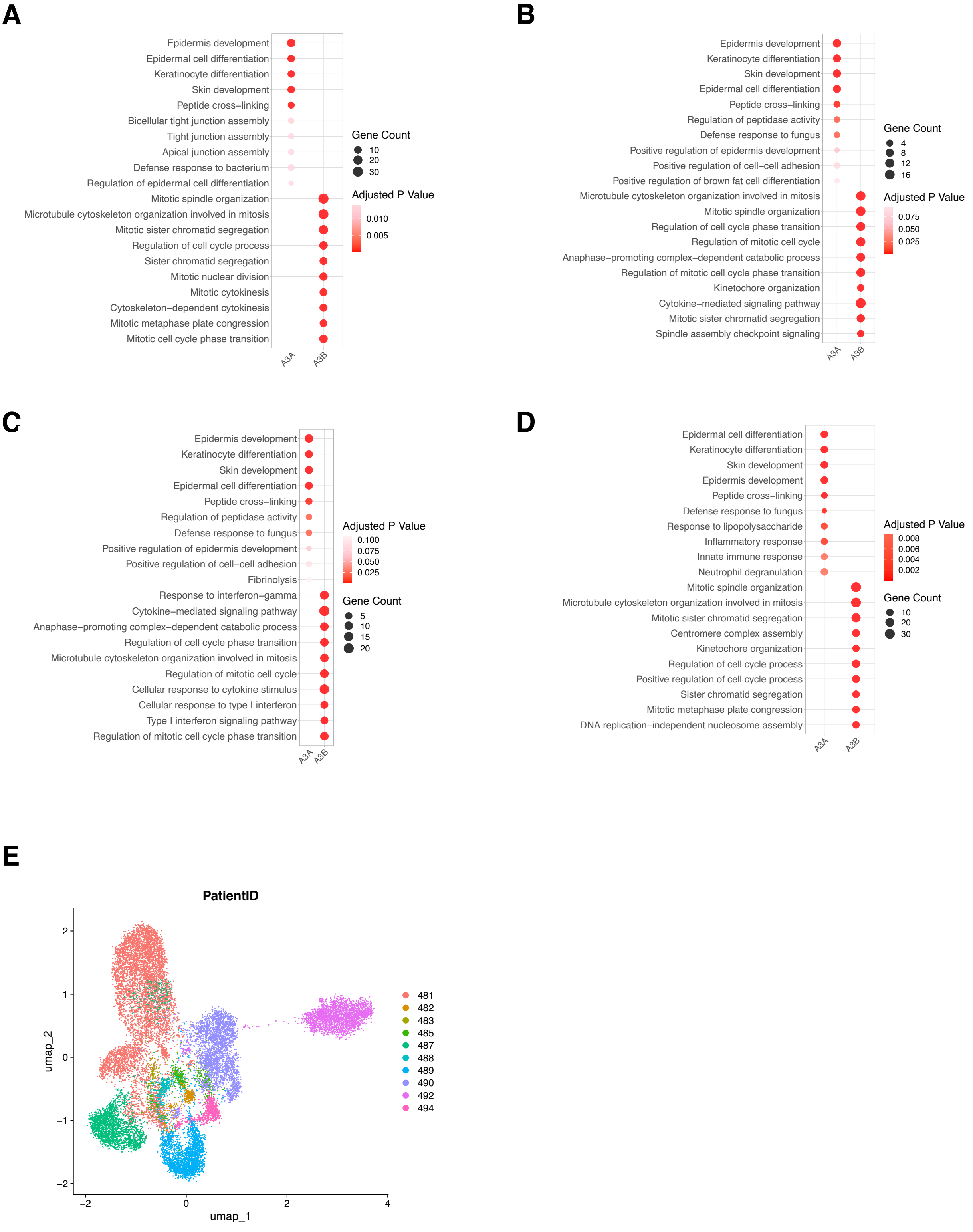

Appendix Figure S9. HNSCC and ESCC display similar expression patterns of *APOBEC3A* and *APOBEC3B* to those observed in the healthy tonsil epithelium. Top 10 GO biological pathways related to the top 100 genes co-expressed with *APOBEC3A* or *APOBEC3B* for datasets **A)** HNSCC (Southampton), **B)** HNSCC validation dataset 1 (Kürten *et al*. 2021), **C)** HNSCC validation dataset 2 (Puram *et al*. 2023), and **D)** ESCC validation dataset. **E)** UMAPs showing clustering of the Southampton HNSCC dataset by patient.

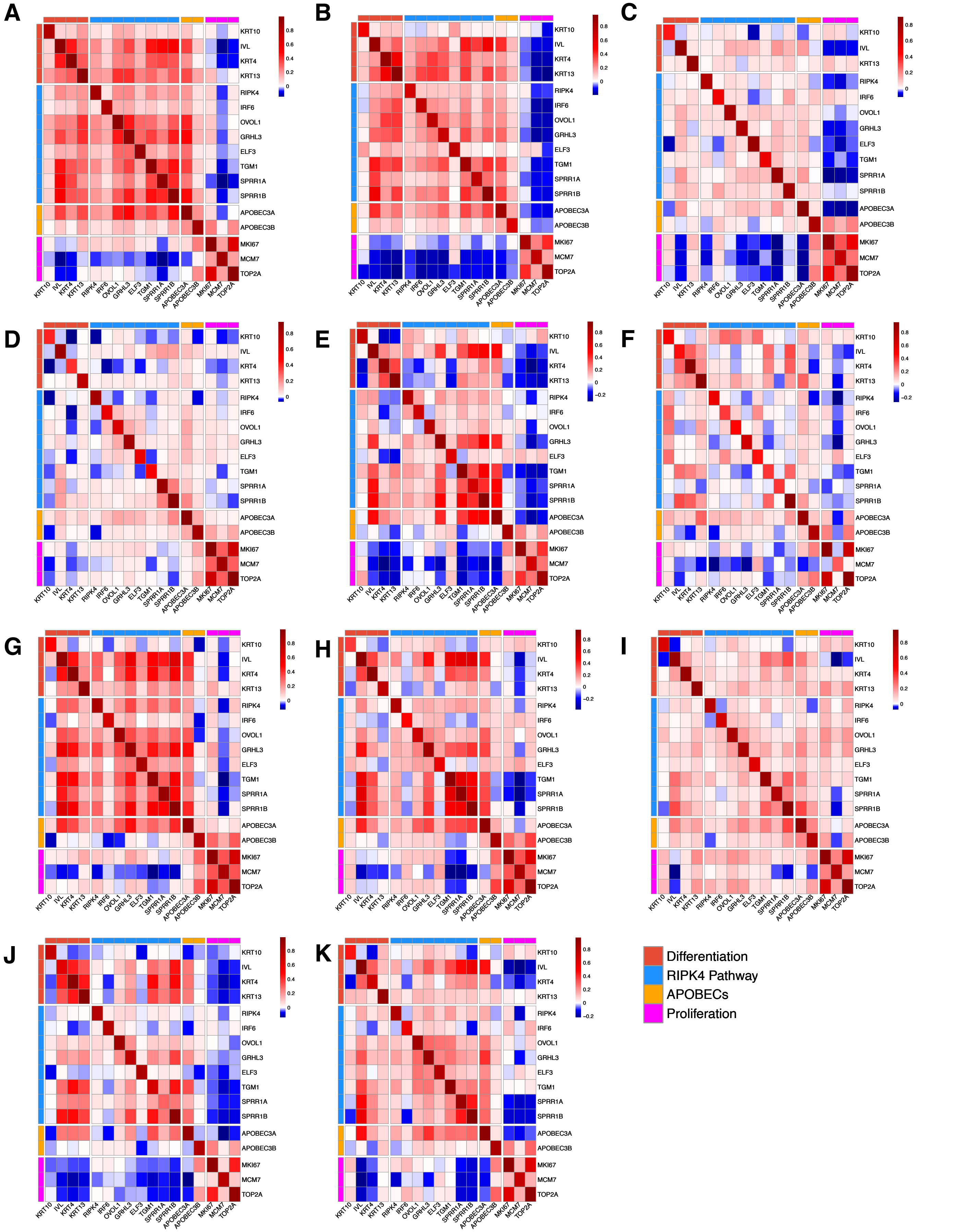

Appendix Figure S10. Co-expression of *APOBEC3A* with differentiation and RIPK4 pathway genes and *APOBEC3B* with proliferation-related genes in the Southampton HNSCC scRNA-seq dataset. Heatmaps showing COTAN co-expression coefficients (red = positively co-expressed, blue = negatively expressed) for **A)** all 10 cases combined and from individual HPV negative tumours **B)** HN481 (7,032 cells), **C)** HN487 (2,129), **D)** HN492 (2,407), and HPV positive tumour patients **E)** HN482 (354), **F)** HN483 (158), **G)** HN485 (592), **H)** HN488 (621), **I)** HN489 (2,411), **J)** HN490 (2,932), and **K)** HN494 (678). Accompanies Figure 5.

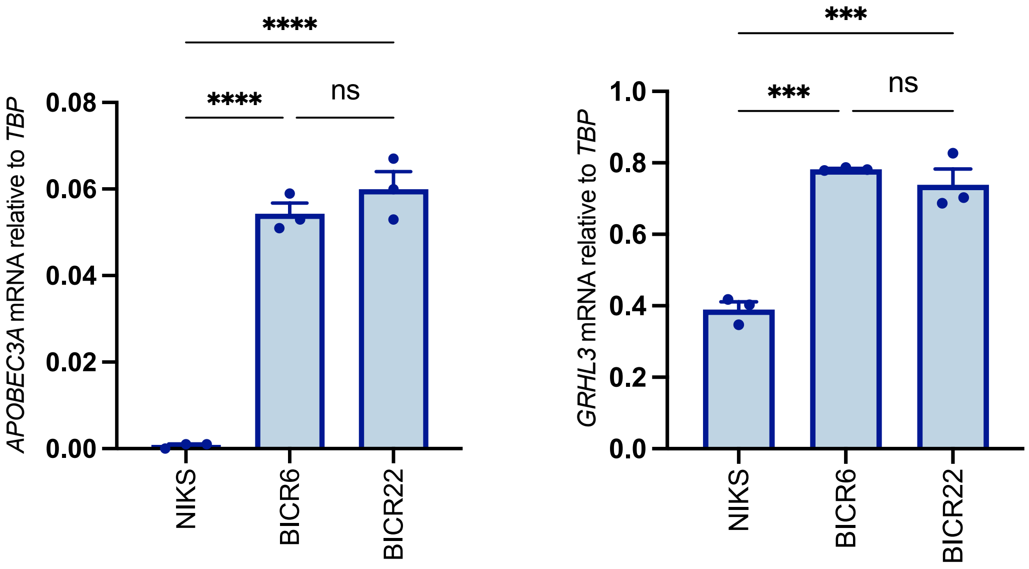

Appendix Figure S11. *APOBEC3A* and *GRHL3* expression in sub-confluent, asynchronous NIKS, BICR6 and BICR22 cell lines. Histograms showing expression of *APOBEC3A* and *GRHL3*, in NIKS, BICR6 or BICR22 cells. Cells were grown to 60-70% confluency and media was replenished 18 hours prior to harvesting. N – 3, * = p-value < 0.05; ** = p-value < 0.01; *** = p-value < 0.001; **** = p-value < 0.0001. One-way ANOVA with Dunnett's multiple comparisons test. Accompanies Figure 5.

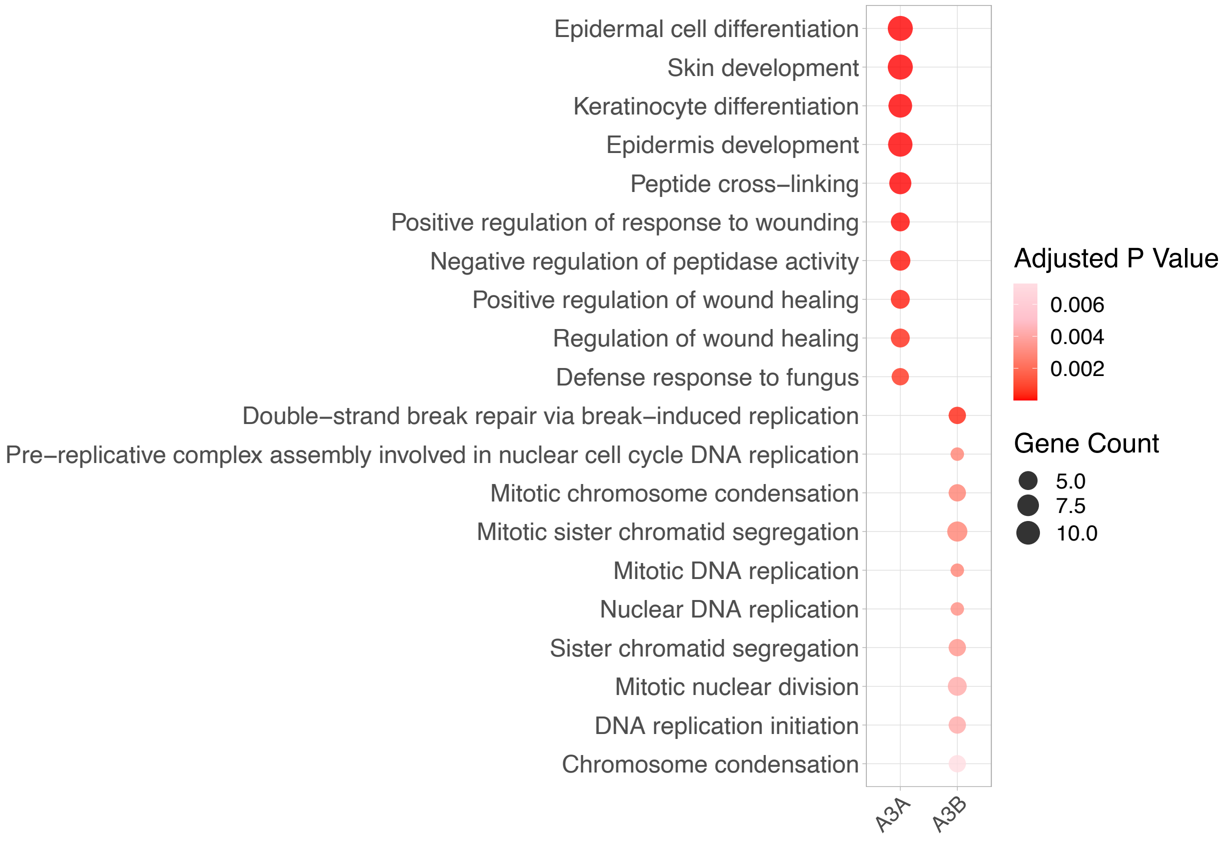

Appendix Figure S12. Pathway analysis of the top 100 genes co-expressed with *APOBEC3A* and *APOBEC3B* in spatial transcriptomic data*.* Top 10 GO biological processes pathways for APOBEC3A and APOBEC3B considering the top 100 genes with the highest correlated expression for each gene.

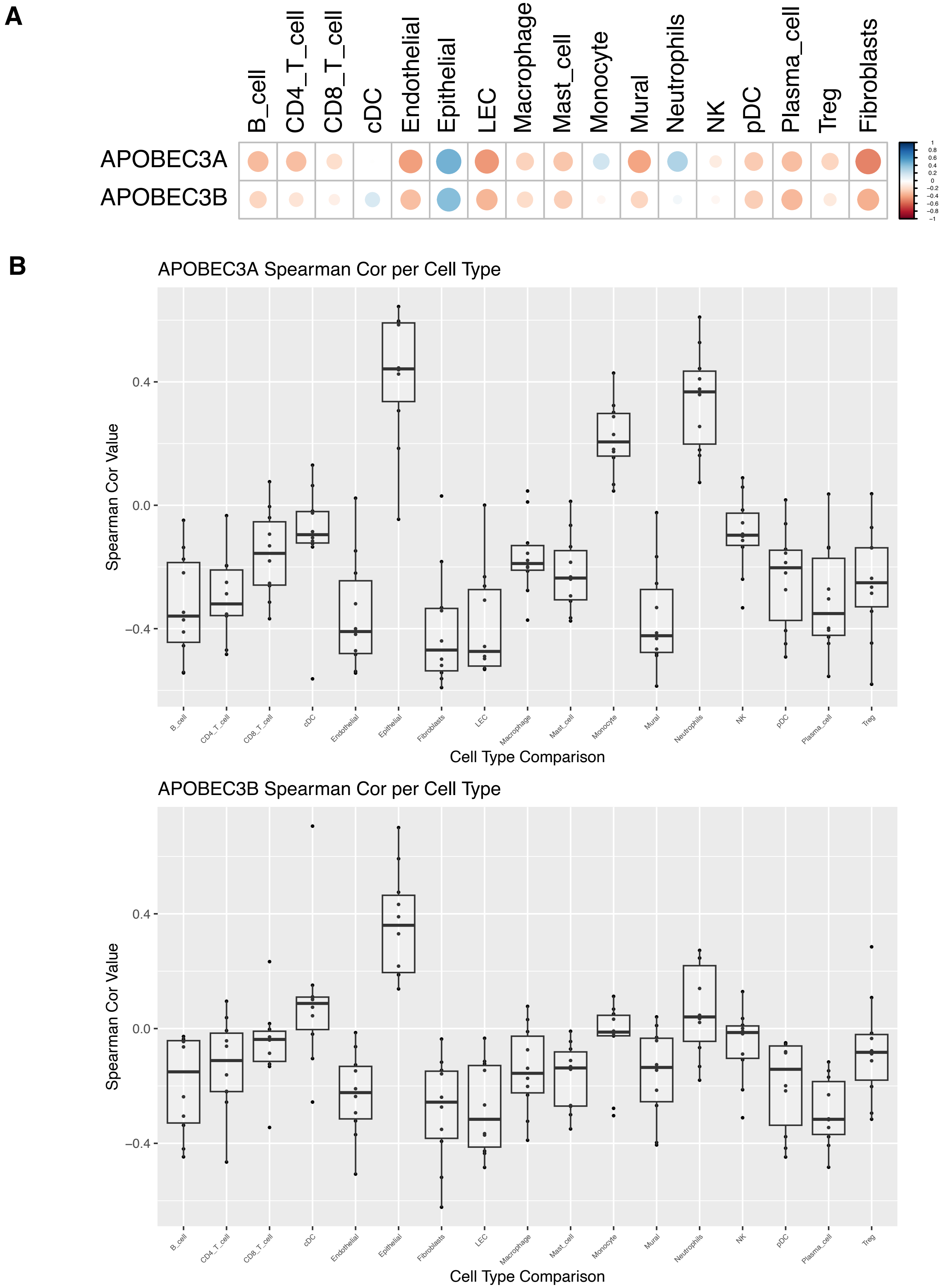

Appendix Figure S13. Deconvolution of spatial transcriptomics data showing the cell types in which *APOBEC3A* and *APOBEC3B* are expressed within each spot. A) Spearman's correlation for *APOBEC3A* and *APOBEC3B* expression and cell-type deconvolution score for every spot in every patient (all correlation coefficients were significant, $p < 0.0001$). **B)** Boxplots showing Spearman's correlations for APOBEC3A (top) and *APOBEC3B* (bottom) expression and cell-type deconvolution score. Each point represents the correlation coefficient value for an individual patient. Accompanies Fig 5.

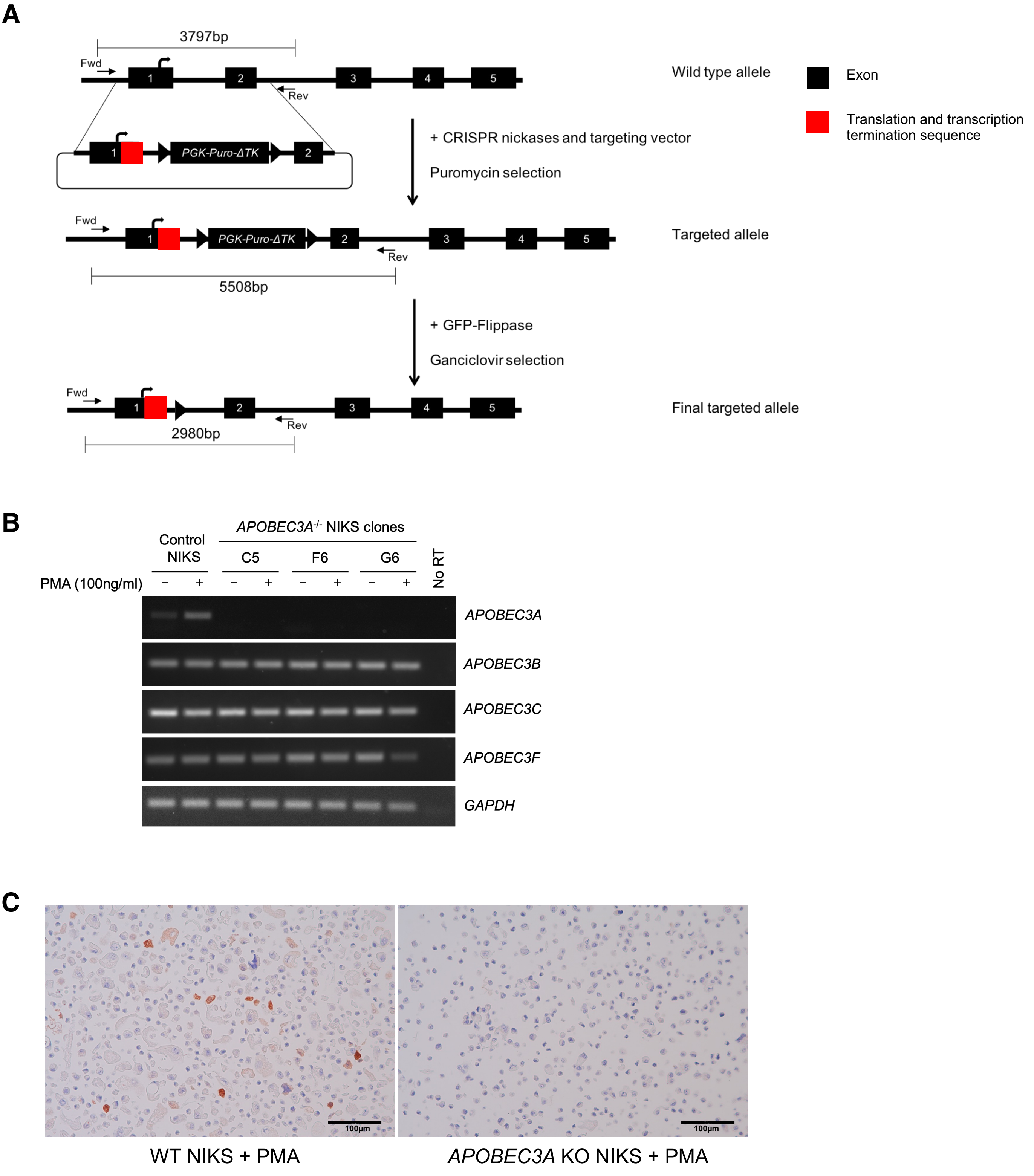

Appendix Figure S14. Generation of *APOBEC3A* knockout (KO) NIKS and specific detection of APOBEC3A by UMN-13 immunohistochemistry. A) Schematic representation of the CRISPR-Cas9-mediated knock-in strategy used to generate APOBEC3A KO NIKS. **B)** Endpoint reverse transcriptase PCR showing levels of *APOBEC3A*, *APOBEC3B*, *APOBEC3C* and *APOBEC3F* mRNA levels in wild-type and APOBEC3A NIKS, -/+ 24h PMA treatment. **C)** Wild-type (WT) or *APOBEC3A* knockout (KO) NIKS (clone C5) were treated with PMA for 24 hours prior to cell harvesting. Cell pellets were paraffin-embedded and formalin-fixed prior to sectioning and staining with anti-APOBEC3A clone UMN-13 as described in Materials and Methods. Accompanies Fig 5 and Fig EV5.

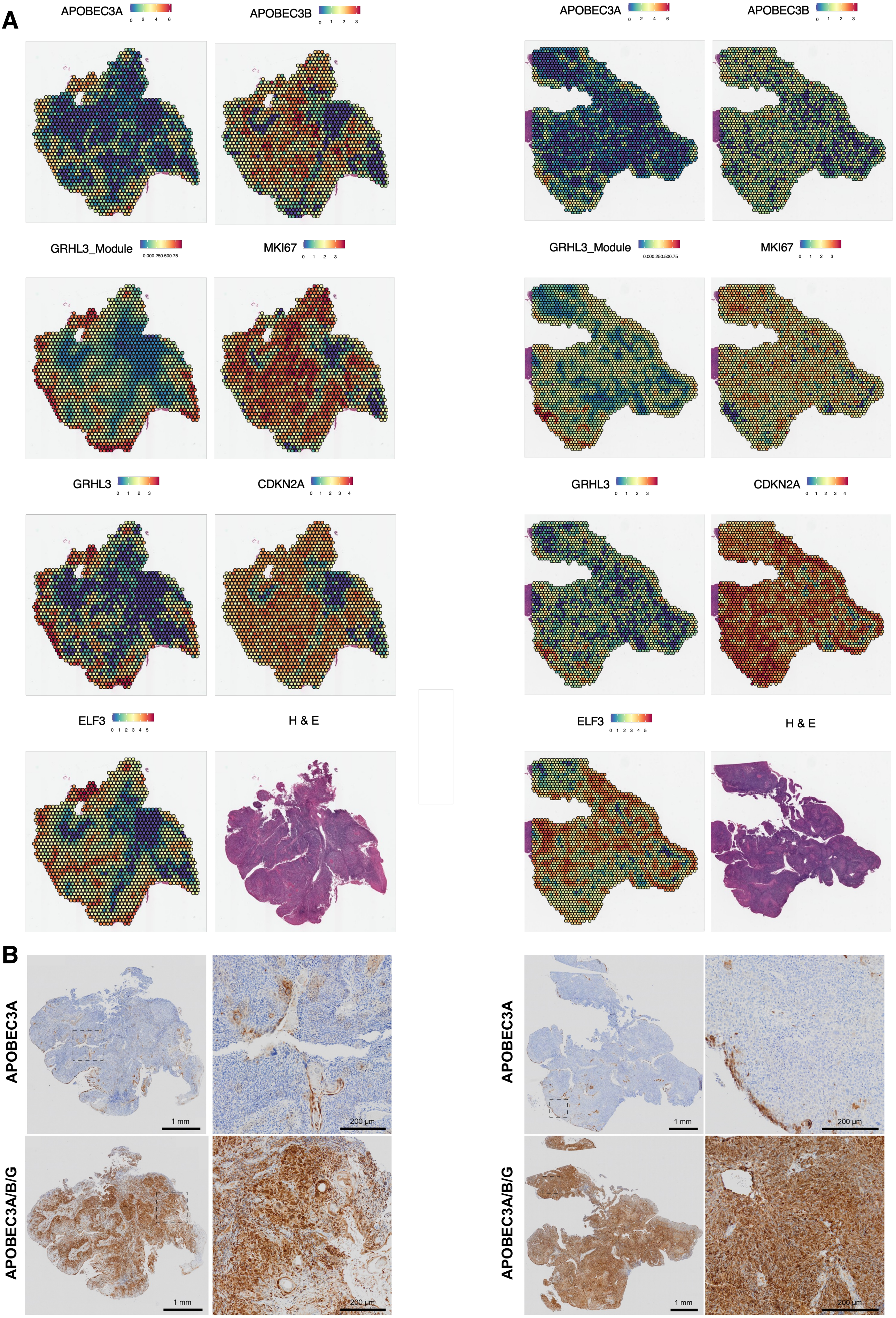

Appendix Figure S15. Analysis of *APOBEC3A* and *APOBEC3B* expression in HNSCC by spatial transcriptomics and immunohistochemistry. (A) Expression patterns of selected transcripts from spatial transcriptomics. **(B)** APOBEC3 protein levels assessed by IHC in two HNSCC cases (HN482 (left) and HN494 (right)). Accompanies Figure 5.
