# Supplementary material for: Differentiation signals induce APOBEC3A expression via GRHL3 in squamous epithelia and squamous cell carcinoma
Source: EMBO J. 2024 Nov 15;44(1):1–29. doi: 10.1038/s44318-024-00298-9 (PMC11696371; doi:10.1038/s44318-024-00298-9)
Supplement: Supplementary file 18 — Expanded View Figures [file 44318_2024_298_MOESM18_ESM.pdf]

Expanded View Figures

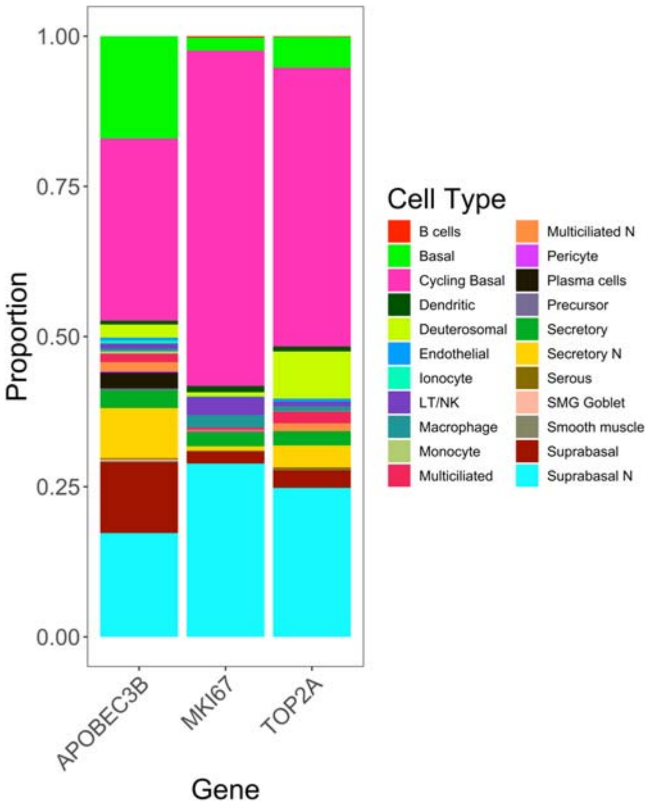

Figure EV1. *APOBEC3B* is predominantly co-expressed with proliferation markers *MKI67* and *MCM7* in cycling basal and suprabasal cells of the healthy human airway.

Stacked barplot showing the proportion of each cell type in the single cell atlas of healthy human airways from Deprez et al that express each selected gene.

© The Author(s)

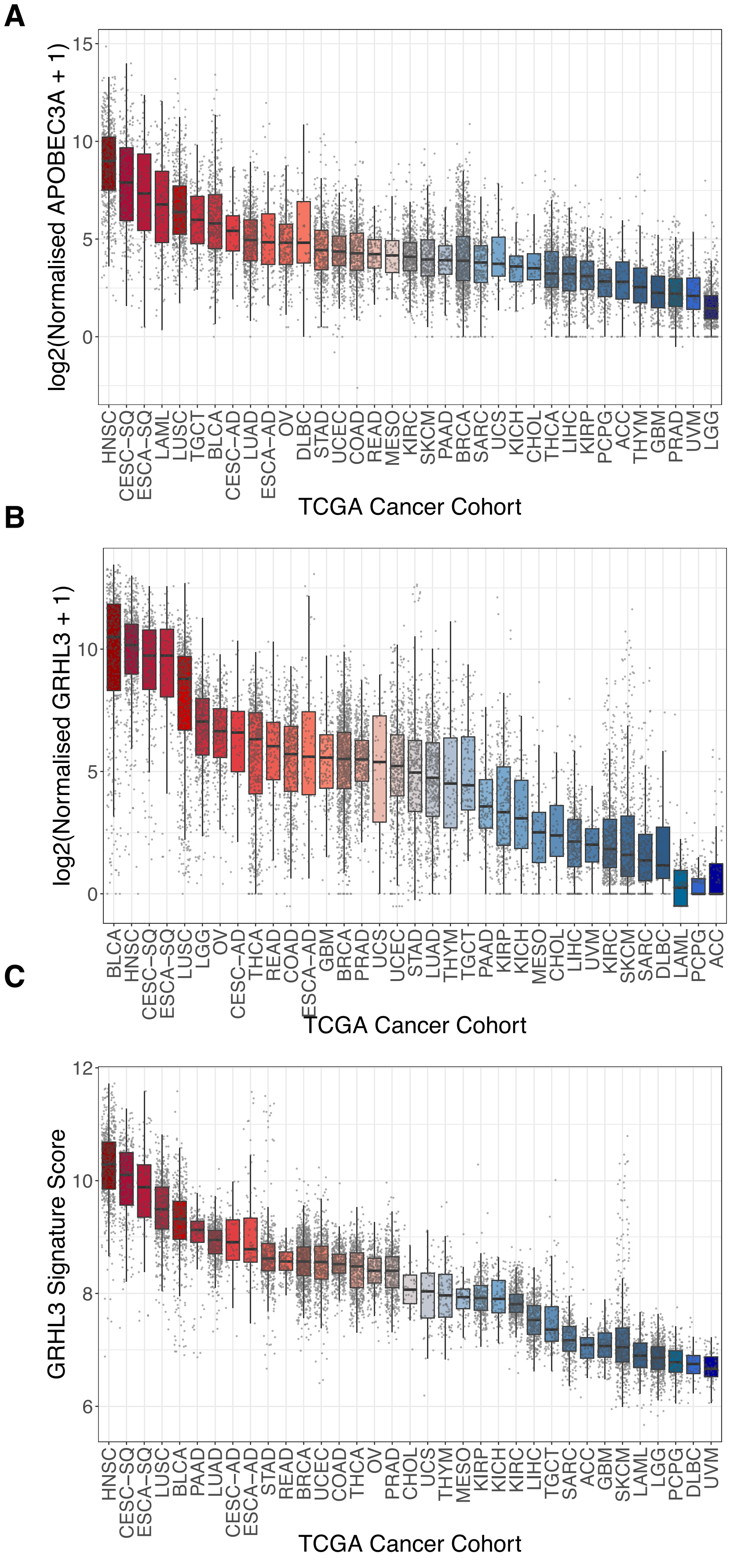

D

TCGA Cancer Type	n	Spearman Correlation	Adjusted p-value
**ESCA-SQ**	**99**	0.65	2.48E-12
**CESC-SQ**	**255**	0.62	2.10E-27
**CESC-AD**	**54**	0.51	2.70E-04
**ESCA-AD**	**97**	0.48	2.17E-06
**THYM**	**122**	0.47	2.15E-07
**HNSC**	**566**	0.43	2.31E-25
**THCA**	**572**	0.40	3.45E-22
**LUSC**	**552**	0.32	9.80E-14
**PRAD**	**550**	0.20	8.56E-06
**BRCA**	**1,215**	0.19	4.07E-10
**CHOL**	**45**	0.15	3.94E-01
**OV**	**308**	0.14	3.29E-02
**BLCA**	**427**	0.12	3.75E-02
**KICH**	**91**	0.10	4.36E-01
**UCEC**	**555**	0.09	7.62E-02
**PAAD**	**183**	0.07	3.94E-01
**SKCM**	**473**	0.06	2.59E-01
**STAD**	**450**	0.06	3.03E-01
**LUAD**	**576**	0.06	2.59E-01
**LAML**	**173**	0.00	9.64E-01
**KIRP**	**323**	0.00	9.64E-01
**DLBC**	**48**	-0.01	9.64E-01
**LIHC**	**423**	-0.01	8.49E-01
**ACC**	**79**	-0.03	8.49E-01
**LGG**	**529**	-0.12	2.05E-02
**SARC**	**265**	-0.12	1.06E-01
**GBM**	**166**	-0.12	1.96E-01
**TGCT**	**139**	-0.13	1.96E-01
**PCPG**	**187**	-0.13	1.30E-01
**READ**	**170**	-0.15	8.67E-02
**COAD**	**492**	-0.16	9.22E-04
**KIRC**	**606**	-0.16	1.75E-04
**MESO**	**87**	-0.23	6.60E-02

E

TCGA Cancer Type	n	Spearman Correlation	Adjusted p-value
**ESCA-SQ**	**99**	0.71	1.2E-15
**CESC-SQ**	**255**	0.68	3.7E-35
**ESCA-AD**	**97**	0.64	5.9E-12
**CESC-AD**	**54**	0.57	9.1E-06
**LAML**	**173**	0.56	3.3E-15
**LUSC**	**552**	0.54	3.9E-42
**THYM**	**122**	0.54	2.9E-10
**HNSC**	**566**	0.54	4.0E-42
**THCA**	**572**	0.53	5.9E-41
**KICH**	**91**	0.46	6.8E-06
**BLCA**	**427**	0.45	8.6E-22
**DLBC**	**48**	0.37	1.2E-02
**SKCM**	**473**	0.37	1.0E-15
**SARC**	**265**	0.34	2.2E-08
**PRAD**	**550**	0.32	4.7E-14
**PCPG**	**187**	0.31	2.9E-05
**GBM**	**166**	0.31	8.6E-05
**LIHC**	**423**	0.30	5.7E-10
**LGG**	**529**	0.28	2.3E-10
**STAD**	**450**	0.27	1.4E-08
**OV**	**308**	0.25	1.5E-05
**LUAD**	**576**	0.24	1.3E-08
**COAD**	**492**	0.24	1.7E-07
**CHOL**	**45**	0.22	1.7E-01
**KIRP**	**323**	0.22	1.1E-04
**UCEC**	**555**	0.18	1.8E-05
**BRCA**	**1,215**	0.17	2.0E-09
**READ**	**170**	0.16	4.6E-02
**PAAD**	**183**	0.07	3.3E-01
**MESO**	**87**	0.04	7.5E-01
**TGCT**	**139**	0.01	9.0E-01
**KIRC**	**606**	-0.07	8.6E-02
**ACC**	**79**	-0.16	1.8E-01

 © The Author(s)

Figure EV2. *APOBEC3A* and *GRHL3* expression by cancer type in TCGA bulk RNA-seq data.

(**A**) Boxplot ranked from highest median (left) to lowest median (right) for *APOBEC3A* gene expression. (**B**) Boxplot ranked from highest median (left) to lowest median (right) for *GRHL3* gene expression. (**C**) Boxplot ranked from highest median (left) to lowest median (right) for GRHL3 signature score based on the mean expression of 127 GRHL3 target genes identified in scRNA-seq SCENIC analysis. Each dot represents an individual tumour sample. (**D**) Spearman correlation coefficients for *APOBEC3A* and *GRHL3* expression in TCGA RNA-seq data. (**E**) Spearman correlation coefficients for *APOBEC3A* expression and GRHL3 signature score in TCGA RNA-seq data. ESCA and CESC were stratified into squamous cell (SQ) and adeno- (AD) carcinomas. For all boxplots, the top, middle and bottom lines of the boxplot represent the upper quartile (Q3), median, and lower quartile (Q1), respectively. The maximum value represented by the top whisker represents the highest observed data point within $Q3 + (1.5 \times (Q3 - Q1))$, and the minimum value represented by the bottom whisker represents the lowest situated point within $Q1 - (1.5 \times (Q3 - Q1))$.

A

B

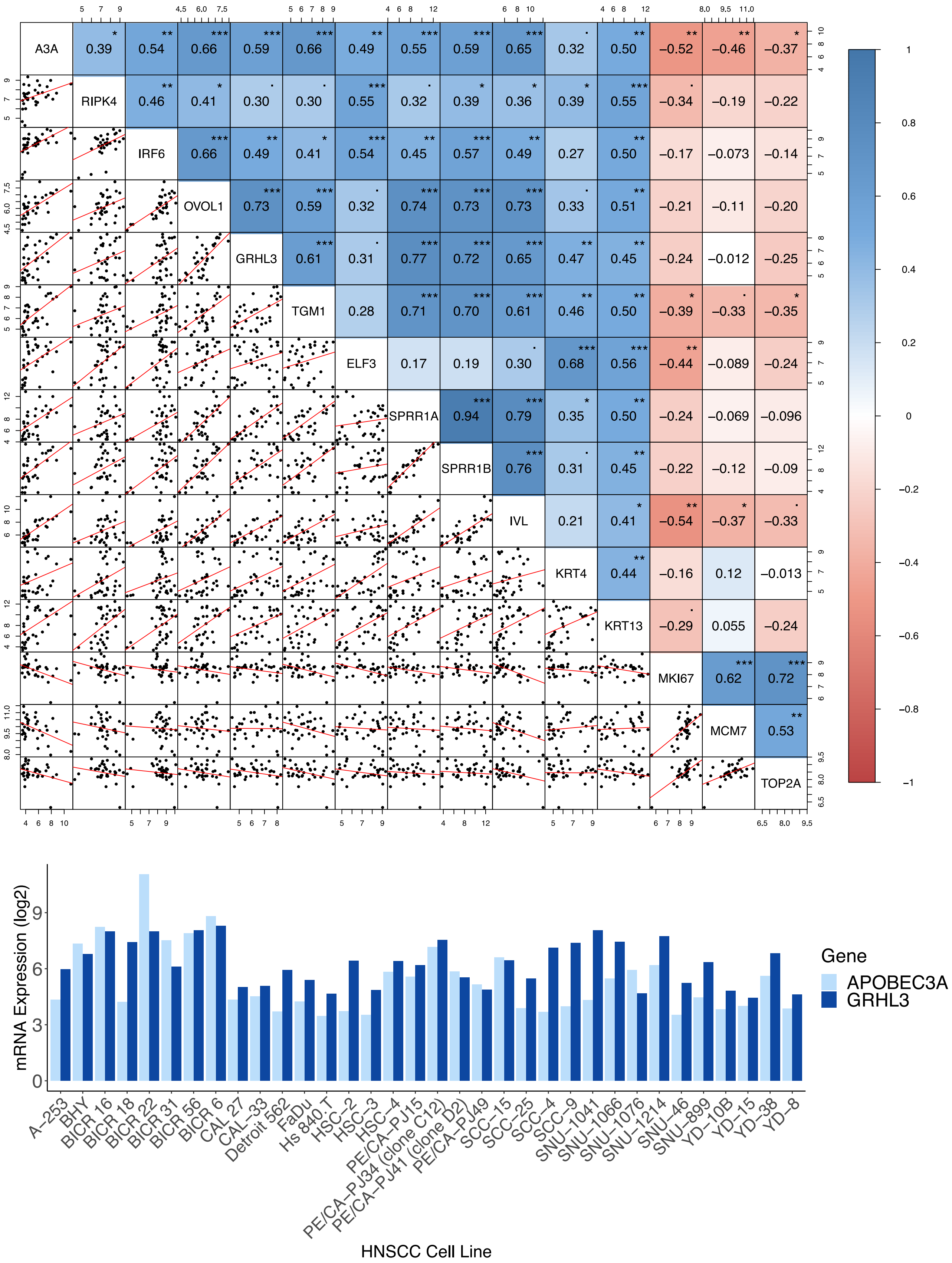

 © The Author(s)

Figure EV3. *APOBEC3A* is positively correlated with differentiation marker genes and negatively correlated with proliferation marker genes in HNSCC cell lines.

(**A**) Spearman's **c**orrelation of *APOBEC3A* expression with the expression of differentiation marker genes, proliferation marker genes, and genes of the RIPK4 pathway in 34 HNSCC cell lines from the CCLE. *P* values calculated by *T*-test: ***$P < 0.0001$; **$P < 0.001$; *$P < 0.05$; $P < 0.1$. (**B**) Log2 expression levels of *APOBEC3A* and *GRHL3* in the 34 individual HNSCC cell lines from the CCLE. Accompanies Fig. 5.

© The Author(s)

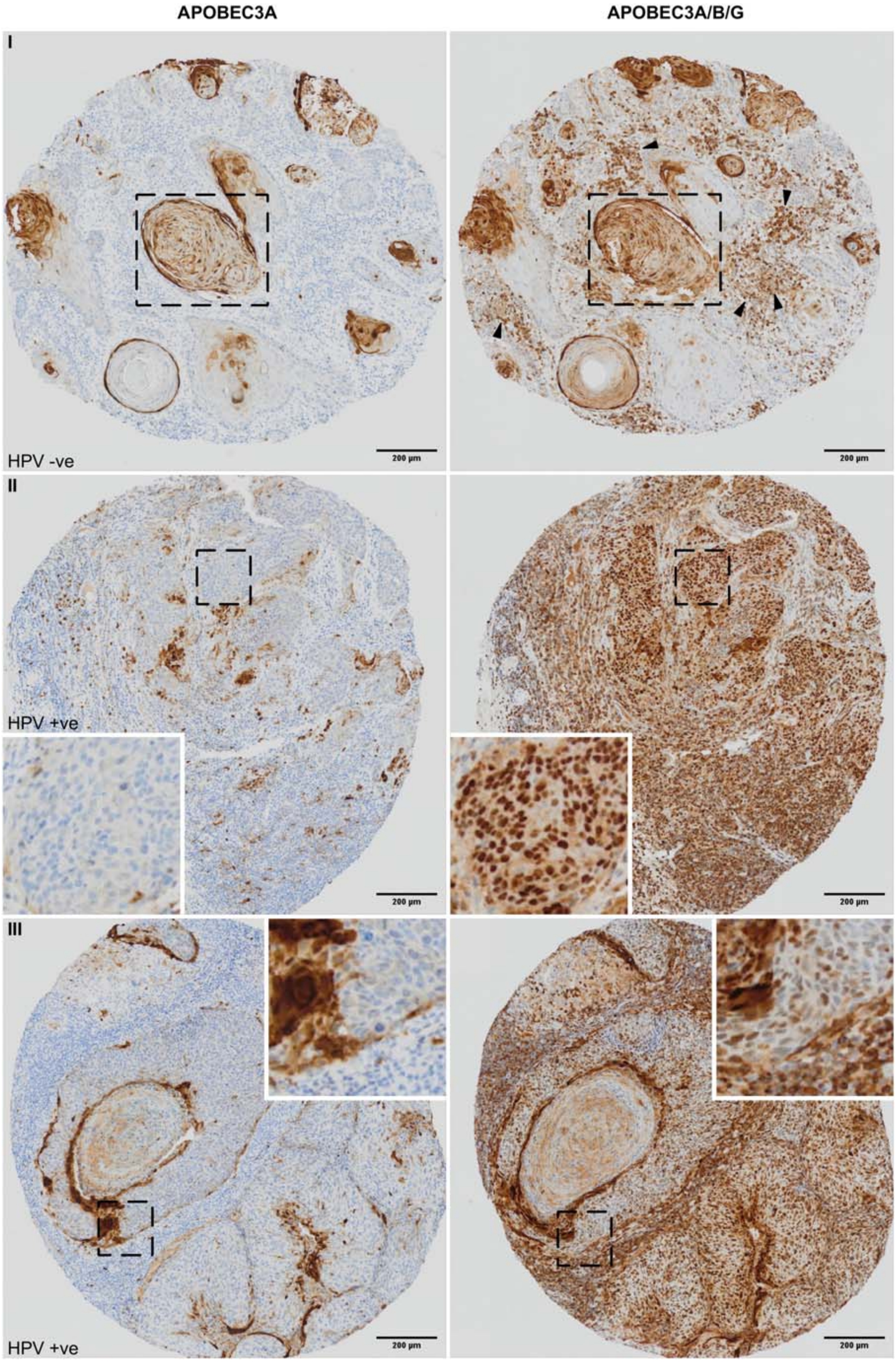

 © The Author(s)

Figure EV4. Detection of APOBEC3A and APOBEC3B protein expression in HNSCC.

Representative images from an HNSCC tissue microarray stained with APOBEC3A-specific (left panels) or APOBEC3A/B/G-specific (right panels) antibodies. Accompanies Fig. 5.
